# The Antiproliferative Activity of Kinase Inhibitors in Chronic Myeloid Leukemia Cells Is Mediated by FOXO Transcription Factors

**DOI:** 10.1002/stem.1748

**Published:** 2014-08-18

**Authors:** Francesca Pellicano, Mary T Scott, G Vignir Helgason, Lisa E M Hopcroft, Elaine K Allan, Mark Aspinall-O'Dea, Mhairi Copland, Andrew Pierce, Brian J P Huntly, Anthony D Whetton, Tessa L Holyoake

**Affiliations:** a Paul O'Gorman Leukaemia Research Centre, College of Medical, Veterinary & Life Sciences, Institute of Cancer Sciences, University of GlasgowGlasgow, United Kingdom; b Stem Cell and Leukaemia Proteomics Laboratory, School of Cancer and Enabling Sciences, Manchester Academic Health Science Centre, The University of ManchesterManchester, United Kingdom; c Department of Haematology and Wellcome Trust Stem Cell Institute, University of Cambridge, Cambridge Institute for Medical ResearchCambridge, United Kingdom

**Keywords:** Chronic myeloid leukemia, BCR-ABL, FOXO transcription factors, Tyrosine kinase inhibitors, CD34^+^ progenitor cells, Quiescence

## Abstract

Chronic myeloid leukemia (CML) is initiated and maintained by the tyrosine kinase BCR-ABL which activates a number of signal transduction pathways, including PI3K/AKT signaling and consequently inactivates FOXO transcription factors. ABL-specific tyrosine kinase inhibitors (TKIs) induce minimal apoptosis in CML progenitor cells, yet exert potent antiproliferative effects, through as yet poorly understood mechanisms. Here, we demonstrate that in CD34+ CML cells, FOXO1 and 3a are inactivated and relocalized to the cytoplasm by BCR-ABL activity. TKIs caused a decrease in phosphorylation of FOXOs, leading to their relocalization from cytoplasm (inactive) to nucleus (active), where they modulated the expression of key FOXO target genes, such as Cyclin D1, ATM, CDKN1C, and BCL6 and induced G1 arrest. Activation of FOXO1 and 3a and a decreased expression of their target gene Cyclin D1 were also observed after 6 days of in vivo treatment with dasatinib in a CML transgenic mouse model. The over-expression of FOXO3a in CML cells combined with TKIs to reduce proliferation, with similar results seen for inhibitors of PI3K/AKT/mTOR signaling. While stable expression of an active FOXO3a mutant induced a similar level of quiescence to TKIs alone, shRNA-mediated knockdown of FOXO3a drove CML cells into cell cycle and potentiated TKI-induced apoptosis. These data demonstrate that TKI-induced G1 arrest in CML cells is mediated through inhibition of the PI3K/AKT pathway and reactivation of FOXOs. This enhanced understanding of TKI activity and induced progenitor cell quiescence suggests that new therapeutic strategies for CML should focus on manipulation of this signaling network. Stem Cells *2014;32:2324–2337*

## Introduction

Chronic myeloid leukemia (CML) arises when the t(9;22) translocation occurs in a normal hemopoietic stem cell (HSC), generating the Philadelphia (Ph) chromosome. The resulting leukemia is driven by the *BCR-ABL* oncogene, encoding a constitutively active protein tyrosine kinase 1. First line therapies for CML involve the protein tyrosine kinase inhibitors (TKIs) imatinib mesylate, dasatinib, and nilotinib. These agents induce rapid cytogenetic responses (CyR) in the majority of CML patients in chronic phase (CP) 2, but in most cases do not eliminate *BCR-ABL* transcripts, suggesting persistence of residual disease. Indeed, residual disease has now been definitively demonstrated in CML patients in CyR 3 and even in those rare patients who achieve and maintain a complete molecular response 4. These findings, together with the rapid kinetics of recurrence in most patients who discontinue TKIs, suggest the presence of leukemic stem/progenitor cells that are TKI-insensitive 5–8. The mechanism(s) for TKI-insensitivity of CML stem/progenitor cells remain(s) unclear, but may in part be explained by recent data showing that primitive CML cells do not depend on BCR-ABL kinase activity for survival 9,10. However, we and others have shown that although CML stem/progenitor cells are relatively insensitive to apoptosis induction, TKIs do exert potent, reversible, antiproliferative effects on these cells in vitro 4,6,11,12. Assuming these effects are replicated within the bone marrow (BM) microenvironment in patients, then eradication of CML may be made even more difficult as TKIs may activate cellular pathways in vivo that lead to G1 arrest and a protective state of induced quiescence.

BCR-ABL activates multiple signal transduction pathways involved in cell survival and proliferation, including the Forkhead box, subgroup O (FOXO) family of transcription factors (TFs) 13. In normal stem/progenitor cells, FOXOs localize in the nucleus and their transcriptional activity results in cell cycle arrest 14. Loss of FOXOs results in an aberrant increase in reactive oxygen species, a dramatic increase in the proportion of cycling HSCs and eventually in HSC exhaustion 15. A transduction/transplantation mouse model that reproduces CML-like myeloproliferative disease has been used to show that FOXO3a has an essential role in the maintenance of leukemic stem cells 16. In this report, the leukemia-initiating cell population contained predominantly active FOXO3a and their ability to generate the disease was significantly decreased by deletion of FOXO3a. Furthermore, BCL6 has been identified as the critical factor mediating the downstream effects of FOXOs in Ph^+^ stem cells by repressing transcription of Arf and p53 17–19. BCL6 was shown to be repressed in a BCR-ABL-dependent manner and required for maintenance of CML stem cells 20,21. Induction of FOXO3a in cell lines has been shown to inhibit cell cycle progression and to induce apoptosis through tumor necrosis factor-related apoptosis-inducing ligand and p53 pathway activation 22,23. Cell line studies suggest that FOXOs may also play a central role in the antiproliferative effects of TKIs. In several BCR-ABL-expressing cell lines, imatinib exposure resulted in FOXO3a activation and cell cycle arrest 21,24–26. However, the role of FOXO TFs on the antiproliferative effects of TKIs in primary CML has not been determined.

Here, we have investigated the mechanism through which TKIs lead to G1 arrest in vitro in primary CD34^+^ CML cells and in vivo in the SCLtTA/BCR-ABL mouse model of CML 27. We propose that by understanding the mechanism of TKI-induced antiproliferative activity, it may be possible to optimize targeting of CML stem/progenitor cells in patients, by preventing or reversing the induced G1 arrest caused by FOXO reactivation and forcing these cells into cycle and toward apoptosis.

## Materials and Methods

### Reagents

Rapamycin and LY294002 were from Calbiochem (Nottingham, U.K.), imatinib, dasatinib, and nilotinib were from Bristol-Myers Squibb (Princeton, New Jersey, USA) and Selleckchem (Newmarket, Suffolk, UK).

### Cell Culture

Primary samples were obtained with written informed consent from patients with newly diagnosed CP CML or patients negative for BM involvement upon lymphoma staging (normal control stem cells). Samples were enriched for CD34^+^ cells using CliniMACS and cultured as previously described 28.

### TKI Treatment of SCLtTA/BCR-ABL Mice

BM from SCLtTA/BCR-ABL mice (*n* = 3) was transplanted intravenously into FVBN recipient mice (*n* = 6). By removing tetracycline from the drinking water, BCR-ABL was induced for 18 days 27. Peripheral blood (PB) was analyzed by fluorescence-activated cell sorting (FACS) after 7 and 15 days of induction to assess leukemia development. After 18 days, half the mice were treated by oral gavage for 6 days with 50 mg/kg per day of dasatinib and the other half with correspondent vehicle. The mice were euthanized and BM c-Kit^+^ cells isolated using c-Kit microbeads and MACS Cell-Separation kit (Miltenyi, Surrey, U.K.).

### RNA Extraction and Real-Time Quantitative PCR

An RNA Extraction Kit was used (Qiagen, West Sussex, U.K.) and cDNA synthesized using a High Capacity cDNA Reverse Transcription Kit (Applied Biosystems, Warrington, U.K.). Alternatively, 300 cells were sorted and processed with “Cells direct one-step q RT-PCR” (Invitrogen, Paisley, UK). Real-time quantitative PCR (Q-PCR) was carried out using FAM-MGB probes for the genes of interest on the ABI7900 (Applied Biosystems) and Fluidigm platforms (Fluidigm Corporation, San Francisco, CA).

### Western Blotting

Cell lysates were subject to Western blotting as previously described 29. Antibodies used were anti FOXO3a, beta-Tubulin, Actin, and GAPDH (Cell Signaling, Hitchin, U.K.). Active Motif Nuclear Extract kit (Rixensart, Belgium) was used according to the manufacturer's instructions.

### Immuno-fluorescence

CD34^+^, K562, and murine BM c-Kit^+^ cells were fixed with 3.7% (wt/vol) formaldehyde/PBS and permeabilized in 0.5% (wt/vol) Triton X-100 for 20 minutes and blocked with 0.2% (wt/vol) gelatin in PBS. Primary antibodies were applied overnight and secondary FITC (Sigma-Aldrich Company, Ltd., UK) antibody for 1 hour at RT. Primary antibodies were anti FOXO1, FOXO3a, FOXO4, p-FOXO1 (Ser319), and p-FOXO4 (Ser193) (Cell Signaling). Nuclei were stained with 4′6-diamidino-2-phenylindole (DAPI) (Vectashield, Burlingame, CA). Images were analyzed with Confocal Zeiss-Axio Imager M1 fluorescence microscope (Carl Zeiss, Jena, Germany). Images were subject to deconvolution (AxioVision software; Carl Zeiss). Fluorescence was quantified using AxioVision software. Three-dimensional fluorescent was measured by Image Processing and Analysis in Java program. Fluorescence in situ hybridization (D-FISH) was performed as previously described 12.

### Flow Cytometry

CD34^+^ cells were resuspended in “Fix and Perm” (Merck Chemicals, Ltd., Nottingham, U.K.). Primary antibodies, FOXO1, FOXO3a, FOXO4, p-FOXO1 (Ser319), p-FOXO3a (Ser253), p-FOXO4 (Ser193), and Cyclin D1 (Cell Signaling), added at RT for 1 hour and secondary for 30 minutes. Cell cycle was analyzed with PI (50 µg/ml) (Sigma-Aldrich Company, Ltd., Dorset, UK), DAPI, 7-AAD, and Ki67 (BD Biosciences, Oxford Science Park, U.K.). Surface markers Annexin V-FITC, CD34-APC, Mac1(CD11b)-PE, and Gr1-APC (BD Biosciences) were added at RT for 15 minutes.

### CD34^+^, K562, and KCL22 Cell Transfections

For transient transfections, pECE-FOXO3aWT or FOXO3aTM (Addgene, Cambridge, MA) containing wild-type FOXO3a (FOXO3a WT) or a mutant form insensitive to AKT phosphorylation (FOXO3a TM) (T32A/S253A/S315A) 30, respectively, were electroporated into Ph^+^ K562, KCL22, or primary CD34^+^ cells using the Amaxa Nucleofector Kit V (Lonza, Wolverhampton, UK) following the manufacturer's instructions. For stable transfections, FOXO3a WT and TM were subcloned into pcDNA 3.1 zeo and transfected into Ph^+^ K562 cells as above and stable cells selected in 200 µg/ml zeocin (Invitrogen). FOXO3a sh-RNA was subcloned from the pLKO.1 puro vector (Open Biosystems) into pLKO.1 GFP vector. The same approach was used to transfect Ph^+^ K562 with pLKO-GFP-FOXO3a and pLKO-GFP-scrambled control. After 24 hours, the cells were sorted based on green fluorescent protein (GFP) expression using a FACS ARIA Flow Cytometer sorter (Becton Dickinson).

### BrdU Proliferation Assay

Prior to harvesting, cells were labeled with BrdU labeling reagent (Roche Applied Science, West Sussex, UK) for 2 hours. The cells were then centrifuged and BrdU incorporation was measured using the Cell Proliferation ELISA (Roche) following the manufacturer's instructions.

### Gene Expression Profiling Using Microarray

RNA extracted from CD34^+^ cells was analyzed with Affymetrix U133A microarray chips (GEO accession number GSE52362). The arrays were performed by Bristol-Myers Squibb (Princeton). Data were RMA normalized and subsequently analyzed using a paired-sample Rank Product 31. Significant results were identified using false discovery rate (FDR) = 0.05. Array normalized data for FOXOs target genes after in vivo TKI treatment of human CML patients were obtained via NCBI GEO (accession GSE12211) 32. Differential expression was identified by LIMMA using *p* < .20.

### Statistical Analysis

Statistical analyses were performed using the Student's *t* test. A level of *, *p* ≤ .05 was taken to be statistically significant. Levels of **, *p* ≤ .01 and ***, *p* ≤ .001 were considered highly statistically significant. Unless otherwise stated, results are given as mean ± SEM.

## Results

### BCR-ABL Induces Phosphorylation and Cytoplasmic Localization of FOXO TFs in Primary CML CD34^+^ Cells

**Figure 1:**
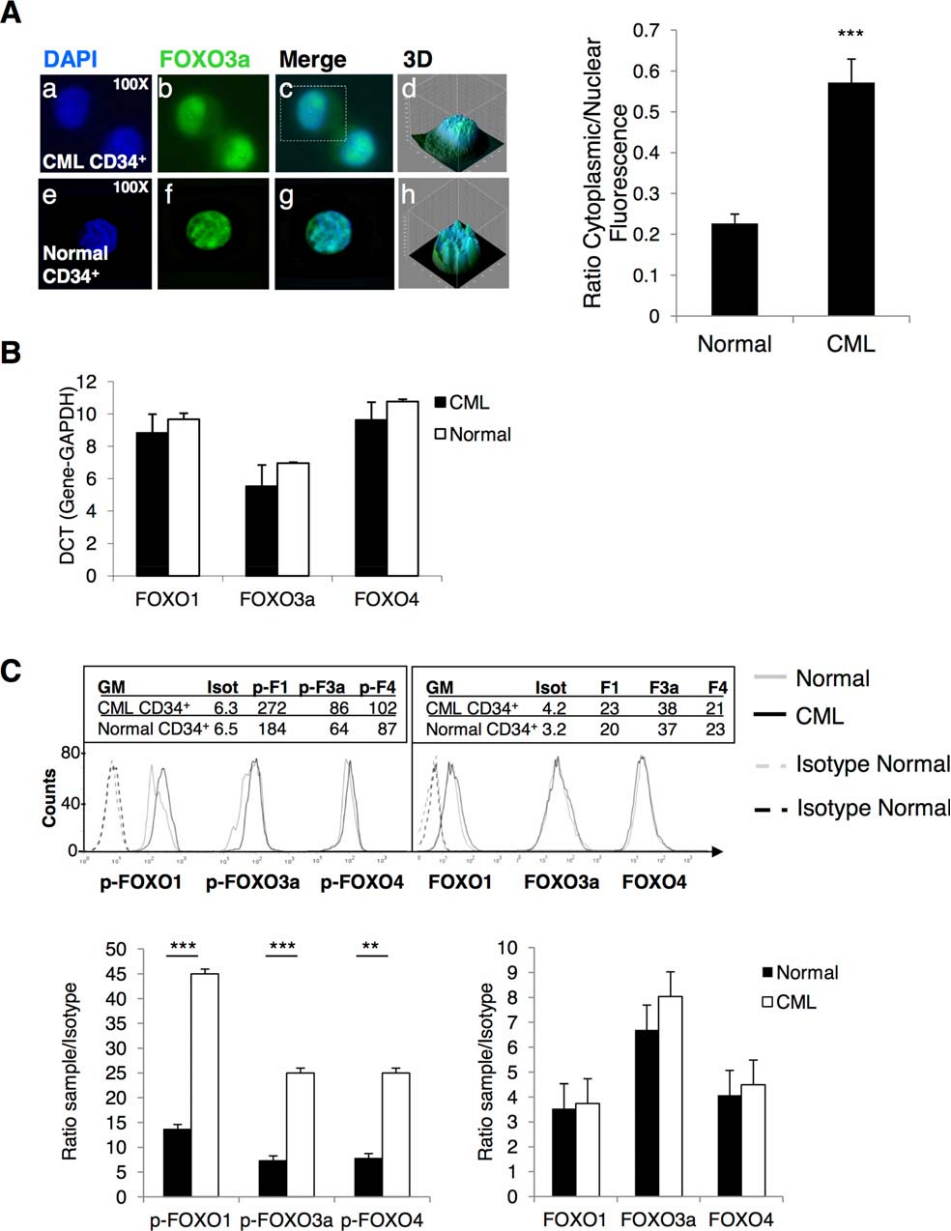
BCR-ABL affects phosphorylation and localization of FOXO isoforms in CML CD34^+^ cells. (**A**, left): Immuno-fluorescence was used to show the localization of FOXO3a in CML (a-d) and normal CD34^+^ cells (e–h) (green: FOXO3a; blue: nuclear DAPI; ×100 magnification). Three-dimensional images for single cells are shown (d and h). (A, right) This was confirmed by quantification of fluorescence intensity in cytoplasm versus nucleus (*n* = 11 and *n* = 37 cells for CML and normal, respectively). **(B):** Levels of FOXO1, 3a, and 4 in CML and normal CD34^+^ cells were measured by Q-PCR (*n* = 3) as shown by the difference in the Ct values (DCT). **(C):** Phosphorylation of FOXO1, 3a, and 4 was measured by flow cytometry in CML and normal CD34^+^ cells (upper panel). Isotype histogram is shown for the three forms of FOXO since the same secondary anti-Rabbit FITC was used to detect all three antibodies. Bar graphs for three independent experiments showing ratio of sample over isotype for total (lower panel, Right) and phosphorylated FOXO1, 3a, and 4 (lower panel, Left) are given (*n* = 3, ***, *p* < .001; **, *p* < .01). Abbreviations: CML, chronic myeloid leukemia; DAPI, 4′6-diamidino-2-phenylindole; GM, geometric mean.

FOXO3a is essential for maintenance of the HSC pool and is likely the key member of the family in regulating quiescence 33. Presently, the precise localization of FOXO in CML progenitor cells is unclear. FOXOs are active in the nucleus, therefore to investigate FOXO3a activity, subcellular localization was performed comparing CP CML and normal progenitor cells (CD34^+^) (Fig. 1A, left). Immuno-fluorescence (IF) showed that FOXO3a was localized both in the cytoplasm and in the nucleus of CML CD34^+^ cells (Fig. 1A, a–d). In contrast, FOXO3a was predominantly nuclear in normal CD34^+^ cells (Fig. 1A, e–h). This was confirmed by relative fluorescence quantification (Fig. 1A, right; ***, *p* < .001).

To analyze whether BCR-ABL expression has effect on the transcription of FOXO1, 3a, and 4, Q-PCR was performed in primary CML and normal CD34^+^ cells. Similar mRNA levels for FOXO1, 3a, and 4 were detected, indicating that BCR-ABL does not affect gene expression but regulates FOXOs at the post-transcriptional level (Fig. 1B).

To determine whether there was a difference in phosphorylation of FOXO1, 3a, and 4 between CML and normal CD34^+^ cells, flow cytometry analysis was performed using antibodies specific for the phosphorylated forms of these FOXOs (Fig. 1C, top panel). The geometric mean (GM) value showed an increase in phosphorylation of FOXO1, 3a, and 4 in CML compared to normal CD34^+^ cells (**, *p* < .01; ***, *p* < .001). No significant difference was detected between CML and normal samples for total FOXO1, 3a, or 4 (Fig. 1C, bottom panels).

### Dasatinib Induces G1 Arrest, Decreases Phosphorylation, and Mediates Relocalization of FOXO1, 3a, and 4 to the Nucleus in Primary CD34^+^ CML Cells

**Figure 2:**
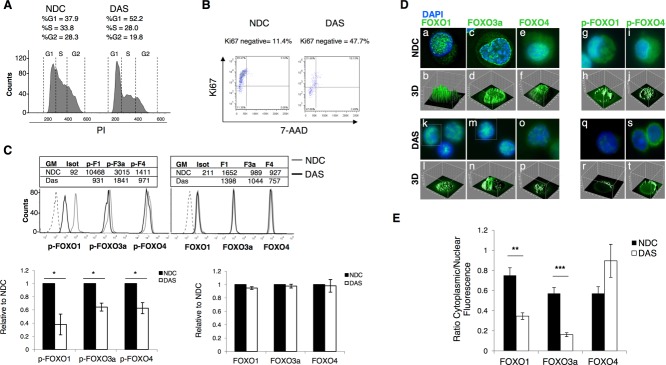
FOXO1, 3a, and 4 phosphorylation are affected by TKI treatment in CD34^+^ chronic myeloid leukemia (CML) cells. **(A):** CD34^+^ CML cells were treated with 150 nM DAS for 24 hours and cell cycle was analyzed by flow cytometry after PI staining (representative plot, *n* = 4) **(B)** Costaining with Ki67/7-AAD was carried out to determine the proportion of quiescent, Ki67-negative cells (representative plot, *n* = 3). **(C):** Levels of pFOXO1, 3a, and 4 (left, lower panel) (*n* = 4, *, *p* ≤ .05), and total FOXO1, 3a, and 4 (right, lower panel) (*n* = 4) following treatment of CD34^+^ CML cells with 150 nM DAS for 24 hours, as measured by flow cytometry. Representative flow cytometry histograms are shown in the upper panel. **(D):** CD34^+^ CML cells were either left untreated (a, c, e, g, and i) or treated with 150 nM DAS for 24 hours (k, m, o, q, and s). Localization of FOXO1, 3a, and 4 and of p-FOXO1 and 4 was analyzed by immuno-fluorescence (overlay image is shown. Green: FOXOs; blue: nuclear DAPI; ×100 magnification). Three-dimensional images of the fluorescence intensity are shown (b, d, f, h, j and l, n, p, r, t). **(E):** Quantification of cytoplasmic versus nuclear fluorescence for total FOXO1, 3a, and 4 (*n* = 6–27 cells, **, *p* < .01 and ***, *p* < .001, respectively). Abbreviations: DAS, dasatinib; NDC, no drug control.

We have previously shown that when CML CD34^+^ cells are exposed to TKIs in vitro this leads to increased numbers of quiescent CD34^+^ CML cells compared to untreated cells 11,12,34. This accumulation of quiescent cells suggests a potent antiproliferative activity of TKIs and, if replicated in vivo, would actually protect CML progenitor cells from the majority of anticancer agents. Dasatinib was selected for these studies as one of the most potent of the TKIs. The antiproliferative activity of TKIs against CD34^+^ CML cells was confirmed by cell cycle analysis, where treatment with 150 nM dasatinib for 24 hours caused a decrease of cells in S phase (from 33.8% to 28%, *, *p* ≤ .05) and an increase in G1 phase (from 37.9% to 52.2%, *, *p* ≤ .05) as shown by representative histogram (Fig. 2A). An induction of quiescence was shown by staining with Ki67/7-AAD which demonstrated an increase in Ki67 negative cells from 11.4% to 47.7% (*, *p* ≤ .05) (Fig. 2B). To examine effects on phosphorylation of FOXO TFs, CD34^+^ CML cells were treated with dasatinib for 24 hours and flow cytometry performed. Significant decreases in phosphorylation of FOXO1, 3a, and 4 in response to dasatinib were observed as indicated by the relative GM (Fig. 2C, representative histogram in top left and for *n* = 4 samples in bar graph, bottom left; *, *p* ≤ .05). Levels of total FOXO1, 3a, and 4 did not change after dasatinib treatment (Fig. 2C, representative histograms top right and *n* = 3 samples in bar graph, bottom right; *, *p* > .05).

Since dasatinib decreased the phosphorylation of FOXO1, 3a, and 4, we investigated whether it also caused relocalization. CD34^+^ CML cells were treated with 150 nM dasatinib for 24 hours, at which time IF showed relocalization of total FOXO1 and 3a from cytoplasm (Fig. 2D, a–f) to nucleus (Fig. 2D, k–p), while little effect was seen with FOXO4. Relocalization of FOXO3a being most evident. Although, FOXO1, 3a, and 4 were located in both nucleus and cytoplasm at baseline, further localization of FOXO1 and 3a to the nucleus was observed after treatment. The phosphorylated form of FOXO1 also decreased in the cytoplasm after treatment, while little change was seen with p-FOXO4 (Fig. 2D, g–j and q–t). The antibody against phospho-FOXO3a was not suitable for IF. Figure 2D shows representative images and replicate experiments are reported in Supporting Information Figure S1. To confirm relocalization of total FOXO1 and 3a, the ratio between cytoplasmic and nuclear fluorescence intensity was quantified (Fig. 2E; **, *p* < .01; ***, *p* < .001). The ratio for FOXO4 showed no significant difference.

### Dasatinib Modulates Expression of Genes that are Downstream Targets of FOXO TFs

**Figure 3:**
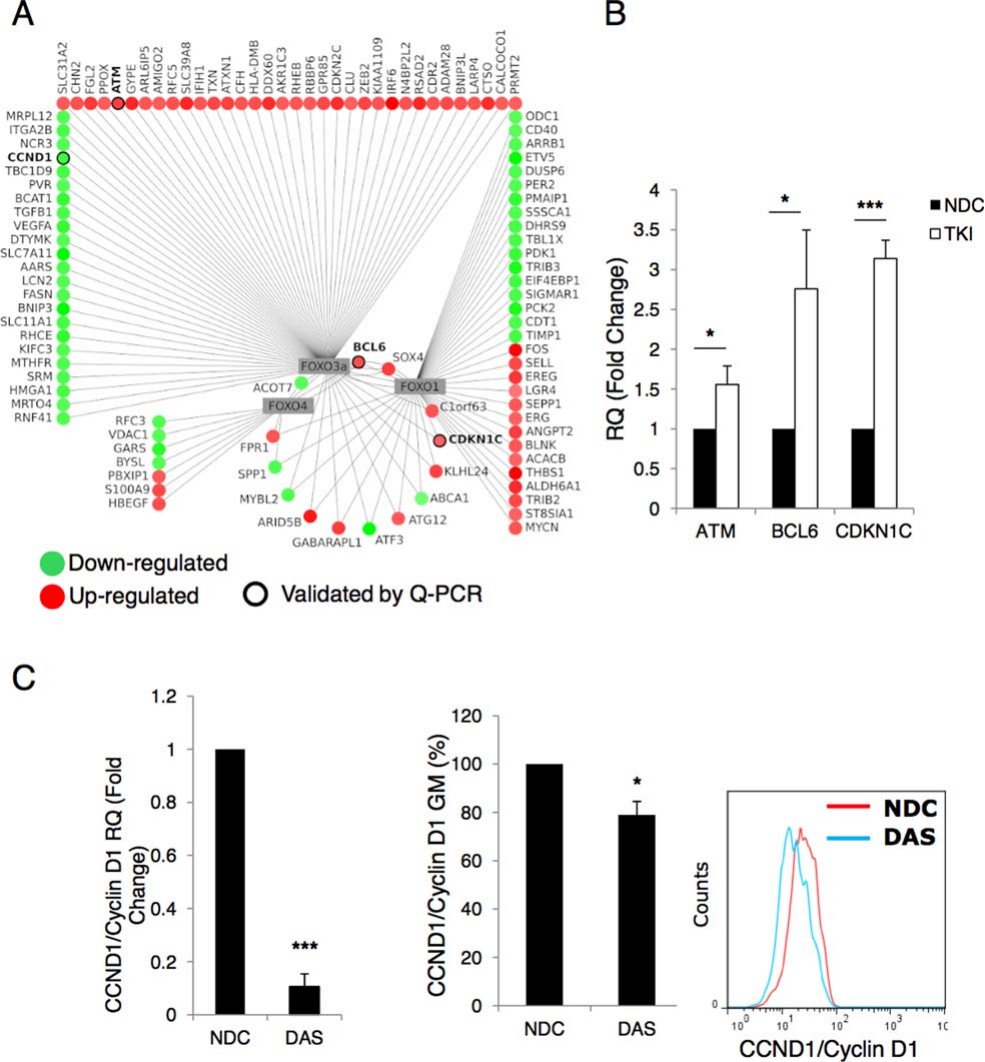
Expression of FOXO target genes are altered following TKI treatment. **(A):** Analysis of an Affymetrix U133A microarray chips showing downstream targets of FOXO1, 3a, and 4 which were significantly modulated following 16 hours exposure to 150 nM DAS in CD34^+^ cells. CCN1D/Cyclin D1, BCL6, p57/CDKN1C, and ATM are highlighted. Interactions were taken from MetaCore knowledge base (22/08/13). **(B):** CD34^+^ CML cells were treated with TKIs (pool of samples treated with imatinib 5 µM, dasatinib 150 nM, and nilotinib 5 µM) for 24 hours and mRNA levels of ATM, BCL6, and p57/CDKN1C were measured by Q-PCR (three independent samples were analyzed in triplicate, ***, *p* < .001; *, *p* ≤ .05). **(C):** Levels of CCN1D/Cyclin D1 mRNA and protein were analyzed after 24 hours of 150 nM DAS treatment. A representative flow cytometry histogram is shown for Cyclin D1 protein (*n* = 3, ***, *p* < .001; *, *p* < .05). Abbreviations: DAS, dasatinib; NDC, no drug control; Q-PCR, quantitative PCR; TKI, tyrosine kinase inhibitor.

To elucidate the mechanism through which TKIs, and FOXO reactivation, induce quiescence in primary CML cells, we interrogated a microarray dataset carried out for CML CD34^+^ cells treated with 150 nM dasatinib for 16 hours (Fig. 3A). FOXO1 (ratio = 1.73, *p* = 6.041 × 10^−5^) and FOXO3a (ratio = 2.69, *p* = 2.394 × 10^−12^) were both identified as connected to genes deregulated by dasatinib treatment (by MetaCore TF analysis). Normalized data were analyzed using Rank Products 31 and differentially expressed genes were identified using a FDR of 0.05. Figure 3A shows genes predicted to be downstream targets of FOXO TFs (extracted from MetaCore KB) which were upregulated (red) or downregulated (green) following treatment.

To validate these results and examine a possible mechanism by which TKIs regulate quiescence through FOXO TFs, the changes in gene expression for a selection of key FOXO target genes (highlighted in black circles) were assayed by Q-PCR (Fig. 3B; *, *p* < .05; ***, *p* < .001). ATM 35 and CDKN1C/p57 36 have both been shown to be essential for maintenance of HSC, while BCL6 has been shown to be essential for leukemic stem cell (LSC) survival 18. Following exposure of CD34^+^ CML cells to TKIs for 24 hours, levels of ATM, BCL6, and CDKN1C/p57 were modulated in the anticipated direction in response to active FOXO TFs and induction of quiescence, pooled results are shown (Fig. 3B).

We then analyzed the transcriptional regulation of CCND1/Cyclin D1 (highlighted in Fig. 3A), a known target of FOXO TFs 14 which is required for cell cycle progression. After 24 hours dasatinib treatment, the mRNA levels of CCND1/Cyclin D1 were dramatically decreased (Fig. 3C). Flow cytometric analysis confirmed the decrease in Cyclin D1 protein although this was more modest (bar graph and representative histogram; *, *p* < .05; ***, *p* < .001).

### TKI Activates FOXO TFs In Vivo

**Figure 4:**
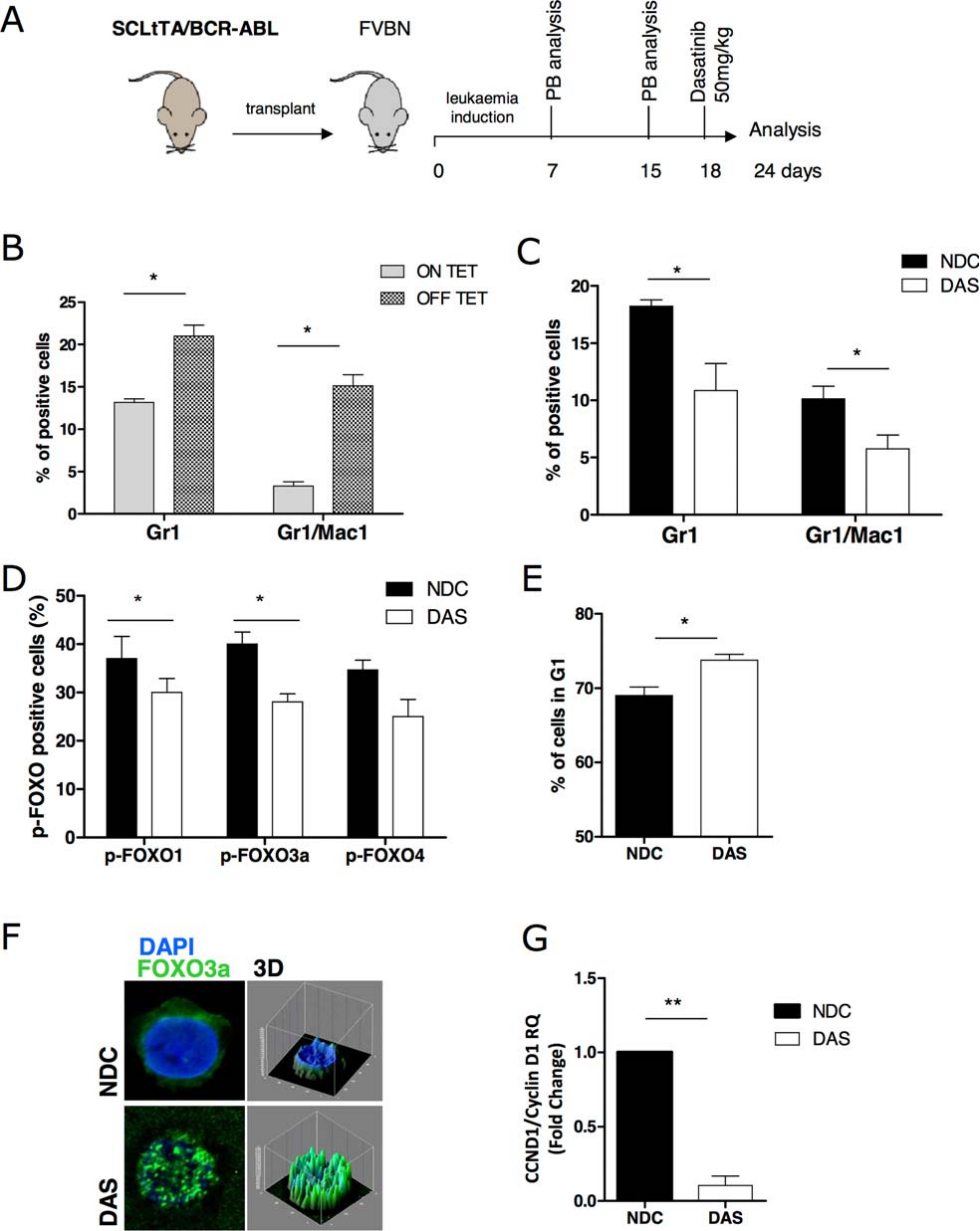
Dasatinib treatment in SCLtTA/BCR-ABL mice induces FOXO1 and 3a activation in vivo. **(A):** Experimental layout for the leukemia induction in SCLtTA/BCR-ABL mice and plan of investigation. **(B):** Percentage of Gr1 and Mac1 in recipient mice after induction of BCR-ABL expression. **(C):** Percentage of Gr1 and Mac1 in recipient mice after 6 days of treatment with 50 mg/kg of dasatinib. **(D):** Level of phosphorylation of FOXO1, 3a, and 4 in c-Kit^+^ cells derived from dasatinib and untreated control mice. **(E):** Cell cycle analysis by PI staining carried out in c-Kit^+^ cells derived from dasatinib and untreated control mice. **(F):** Localization of FOXO3a analyzed by immuno-fluorescence (overlay image is shown. Green: FOXO3a; blue: nuclear DAPI; ×100 magnification). Three-dimensional images of the fluorescence intensity are shown. **(G):** mRNA levels of CCN1D/Cyclin D1 were measured by quantitative PCR in c-Kit^+^ cells derived from dasatinib and untreated control mice (*n* = 3/arm) (*, *p* < .05; **, *p* < .01). Abbreviations: DAS, dasatinib; DAPI, 4′6-diamidino-2-phenylindole; NDC, no drug control.

To investigate whether TKI-induced G1 arrest was mediated by FOXOs in vivo, we initially interrogated a genome wide array profile carried out on samples derived from CP CML patients treated with imatinib for 7 days (accession GSE12211) 32. We observed that several downstream target genes of FOXOs (in particular of FOXO1 and 4) were regulated in the same direction as observed in our in vitro analysis (Supporting Information Figs. S2, S3A). To support these data, we used a SCLtTA/BCR-ABL inducible mouse model 27. To generate mice with a CML-like disease, total BM cells derived from the transgenic SCLtTA/BCR-ABL mice (*n* = 3) were pooled and transplanted into irradiated FVBN recipient mice (Fig. 4A; *n* = 6). Omission of tetracycline from the drinking water of recipient mice led to BCR-ABL expression in the stem/progenitor cells compartment. The mice were checked for signs of leukemia development after 7 and 15 days by staining with Gr1 and Mac1 (CD11b) myeloid markers. After 18 days, a significant increase Gr1 /Mac1 staining was detected in induced mice compared to control mice (Fig. 4B, *, *p* < .05). Half the cohort was then treated for 6 days with dasatinib (50 mg/kg per day), while the other half received vehicle only. At the end of treatment, the mice were sacrificed and analyzed. A marked decrease in Gr1/Mac1percentage was observed in the PB of treated mice (Fig. 4C, *, *p* < .05). BM cells were enriched for c-Kit^+^ progenitor cells and phosphorylation of FOXO TFs analyzed by flow cytometry. Significant decreases in phosphorylation of FOXO1 and 3a (but not FOXO4) were observed in the c-Kit^+^ progenitor cells from dasatinib treated mice (Fig. 4D, *, *p* < .05). The antiproliferative activity of TKIs against c-Kit^+^ cells was confirmed by cell cycle analysis, where a significant increase in the percentage of cells in G1 phase was observed in dasatinib treated mice (Fig. 4E, *, *p* < .05).

By IF we observed that the dasatinib-mediated decrease in phosphorylation of FOXO3a was accompanied by a relocalization of FOXO3a to the nucleus in c-Kit^+^ cells, suggesting its activation (Fig. 4F). Finally, we analyzed the transcription of CCND1/Cyclin D1 and found it significantly decreased in the cells derived from treated mice (Fig. 4G, **, *p* < .01), suggesting activation of the FOXO3a signaling pathway. Complementary results were recently observed by Hurtz et al., where upregulation of FOXO3a target gene BCL6 was detected in a leukemia mouse model upon TKI treatment 18.

### Over-Expression of FOXO3a or Inhibition of PI3K/AKT/mTOR Signaling Inhibits Proliferation and Enhances Dasatinib Activity in Ph^+^ Cells

**Figure 5:**
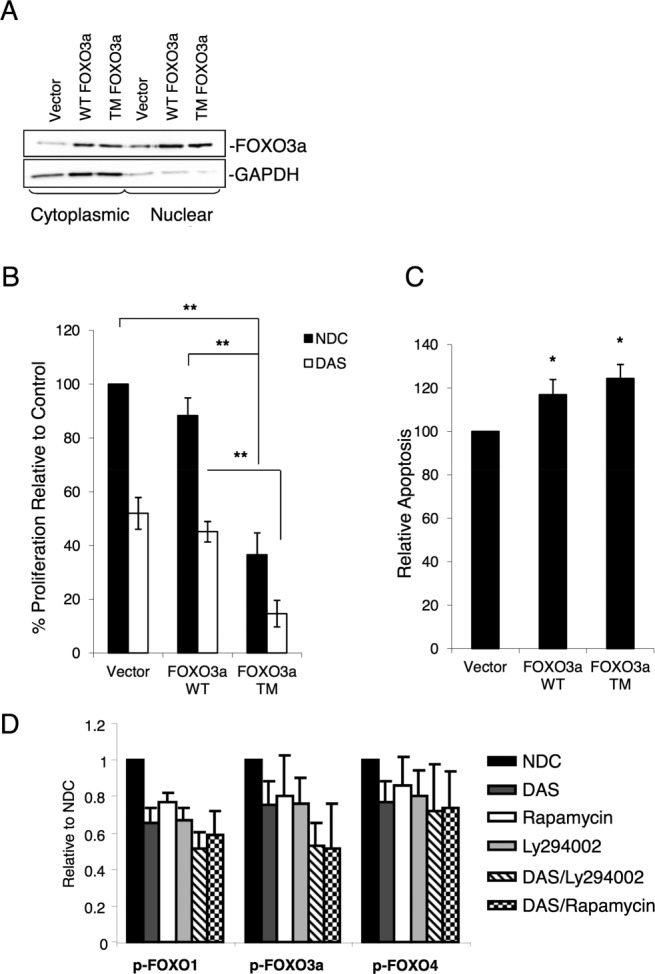
Over-expression of FOXO3a induces strong inhibition of proliferation in the K562 cell line. **(A):** FOXO3a WT, FOXO3a TM (a constitutively active mutant), and vector control were transiently over-expressed in K562 cells and cytoplasmic versus nuclear fractionation carried out. Following isolation, 10 µg of nuclear and cytoplasmic lysates were separated by SDS PAGE and the distribution of FOXO3a determined by Western blotting. GAPDH was used as a cytoplasmic marker. **(B):** Cells transfected with FOXO3a WT or TM were treated or not with dasatinib (DAS, 10 nM) for 24 hours and BrdU used to assess proliferation (*n* = 3, **, *p* < .01). **(C):** Levels of apoptosis were determined by Annexin V/7-AAD staining (*n* = 3, *, *p* ≤ .05). **(D):** CML CD34^+^ cells were treated for 24 hours with rapamycin (10 nM), LY294002 (25 µM), DAS (150 nM), or the combination of DAS with either LY294002 or rapamycin, and FOXO1, 3a, and 4 phosphorylation were measured by flow cytometry (*n* = 3). Statistical analysis for each treatment is reported in Supporting Information Figure S2. Abbreviations: DAS, dasatinib; NDC, no drug control.

To determine whether the antiproliferative activity of TKIs against Ph^+^ cells was mediated by reactivation of FOXO TFs, the CML cell line K562 was used to transiently over-express FOXO3a WT versus a constitutively active mutant form (insensitive to AKT phosphorylation) of FOXO3a (FOXO3a TM). Cells were transfected with FOXO3a WT, FOXO3a TM, or vector alone. Cellular fractionation followed by Western blotting showed that over-expressed FOXO3a mainly localized in the nuclear fractions, GAPDH being used as a cytoplasmic marker (Fig. 5A). A BrdU assay performed on these cells showed inhibition of proliferation in cells transfected with FOXO3a, either WT or TM (Fig. 5B, 12% inhibition with FOXO3a WT and 63% with FOXO3a TM, **, *p* < .01, *n* = 3). The active mutant FOXO3a TM showed a much higher activity in blocking proliferation of K562 cells compared to FOXO3a WT. Indeed FOXO3a TM alone inhibited proliferation to a similar degree as 24 hours incubation with 10 nM dasatinib (used at lower concentration as these cells are more sensitive than primary CD34^+^ CML cells), confirming FOXO3a plays an important role in the cell cycle regulation of these cells. Depending on the cellular context, high levels of FOXO activity can also induce apoptosis. As shown in Figure 5C, induced expression of either FOXO3a WT or TM resulted in modestly enhanced levels of apoptosis (*, *p* < .05). Similar results were seen when we transiently transfected these constructs into a second cell line, KCL22 and CML CD34^+^ cells (Supporting Information Fig. S3A, S3D). In KCL22 cells, FOXO3a TM induced a significant increase in the percentage of cells in G1 which was not further increased by dasatinib (Supporting Information Fig. S3B; *, *p* < .05; **, *p* < .01). Similarly, the FOXO3a TM induced a significant increase in apoptosis in these cells, which again was not enhanced by dasatinib (Supporting Information Fig. S3C; *, *p* < .05; **, *p* < .01). Both the FOXO3a WT and TM mutants increased the percentage of cells in G1 in the CML CD34^+^ cells, again this was not further enhanced by dasatinib in FOXO3a TM-transfected cells (Supporting Information Fig. S3E). The levels of apoptosis however were only slightly enhanced by the constructs (Supporting Information Fig. S3F). In CML, alterations in FOXO activity may not be mediated solely through the BCR-ABL/PI3K/AKT. We have dissected this to some degree via treatment with the mTOR inhibitor rapamycin (mTORC2 is an essential AKT activator for FOXO regulation 37) and the PI3K inhibitor LY294002 to determine whether these drugs were able to inhibit phosphorylation of FOXO1, 3a, and 4 and thus restore FOXO activity in CD34^+^ CML cells. The cells were treated for 24 hours with rapamycin (10 nM), LY294002 (25 µM), dasatinib (150 nM), or the combination of dasatinib with either LY294002 or rapamycin, and then phosphorylation status of FOXO1, 3a, and 4 was measured by flow cytometry (Fig. 5D). *p* values for treatment comparisons are reported in Supporting Information Figure S4. For FOXO1, dasatinib, rapamycin, and LY294002 produced significant decreases in phosphorylation compared to the no drug control, but no differences were detected between the three treatments (*, *p* > .05). The combination of dasatinib with rapamycin or LY294002 further decreased FOXO1 phosphorylation compared to single agent rapamycin or LY294002, respectively, suggesting enhanced inhibition by this combination of inhibitors indicating multiple pathways for activation. A decrease in phosphorylation of FOXO4 was less evident and similar for all the drugs used, including the combinations.

### Over-Expression of FOXO3a Is Sufficient to Induce Quiescence in Ph^+^ Cells

**Figure 6:**
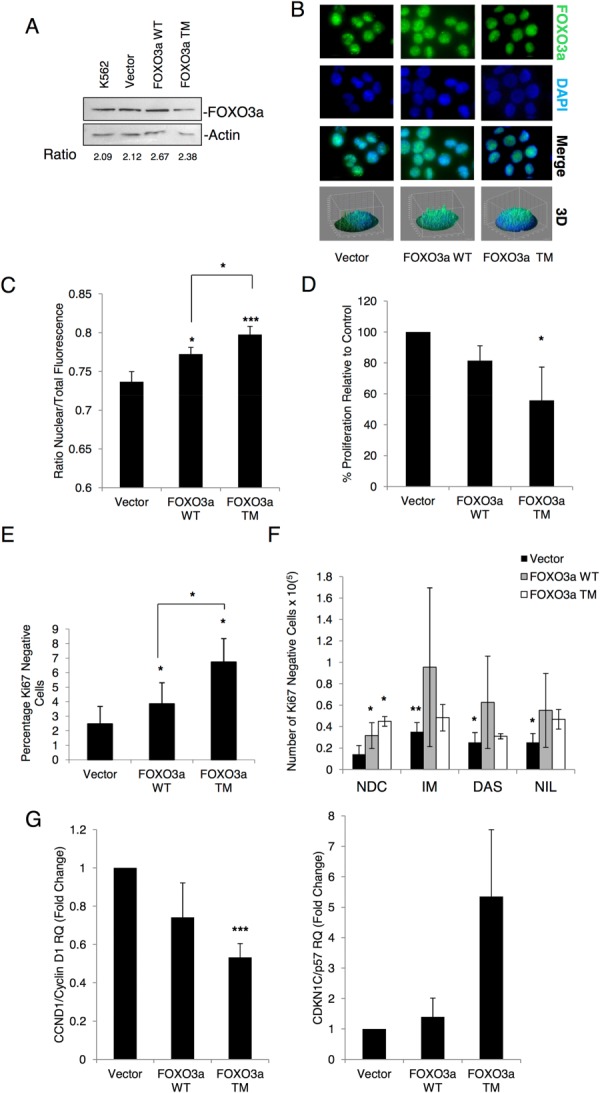
Stable transfection of a FOXO3a active mutant into K562 cells increases quiescence. K562 cells were stably transfected with FOXO3a WT, FOXO3a TM, or an empty vector control. **(A):** Cells were lysed in RIPA buffer and 20 µg of each were separated by SDS-PAGE, Western blotting was used to show levels of FOXO3a and Actin. Fold change relative to Actin is shown. **(B):** Localization of FOXO3a WT and TM was determined by IF (green: FOXO3a, blue: nuclear DAPI, ×100 magnification) and **(C)** quantification of nuclear to total fluorescence (*n* = 31–45 cells, **, *p* < .01, *, *p* ≤ .05). **(D):** BrdU incorporation was used to measure proliferation in stable cell lines (*n* = 3, *, *p* ≤ .05), while **(E)** shows the percentage of Ki67-negative cells as determined by Ki67/7-AAD staining (*n* = 3, *, *p* ≤ .05). To compare this to quiescence induced by TKIs, cell lines were treated for 72 hours with IM (1 µM), DAS (10 nM), or NIL (50 nM) before being stained for Ki67/7-AAD. **(F):** Shows the number of Ki67-negative cells following treatment (*n* = 3, **, *p* < .01; *, *p* ≤ .05 relative to vector only NDC). **(G):** Levels of CCND1/Cyclin D1 (left, *n* = 3 *, *p* < .001) and p57/CDKN1C (right, *n* = 3) mRNA were determined by quantitative PCR. Abbreviations: DAS, dasatinib; DAPI, 4′6-diamidino-2-phenylindole; IM, imatinib; NDC, no drug control; NIL, nilotinib.

To determine whether over-expression of FOXO3a is sufficient to induce elevated levels of quiescence in Ph^+^ cells and could account for the majority of the antiproliferative activity of TKIs, K562 cells were transfected to create stable cell lines over-expressing either FOXO3a WT or FOXO3a TM. By creating stable cell lines we were able to bypass the apoptotic effects of the constructs which we saw in transient transfections. Although the stable cell lines expressed similar levels of total FOXO3a compared to either parental or vector transduced K562 cells (Fig. 6A), both had more nuclear FOXO3a as shown by IF (Fig. 6B). Quantification of nuclear to total fluorescence ratio confirmed levels of nuclear FOXO3a were significantly higher in the WT and TM expressing cells (Fig. 6C, *, *p* < .05; ***, *p* < .001). It is predicted that any cells expressing high levels of FOXO3a would have exceeded the “apoptosis threshold” and died during the selection process. To determine the effect of stable expression of these constructs on cell proliferation, BrdU assay was carried out. As with the transient transfections, the cells expressing the active mutant FOXO3a TM showed a significant decrease in proliferation (Fig. 6D, *, *p* < .05). Moreover, over-expression of FOXO3a WT and TM increased the percentage of quiescent K562 cells, as shown by Ki67/7-AAD labeling, with the percentage of quiescent cells higher for FOXO3a TM versus WT (Fig. 6E, *, *p* < .05). The two over-expressing cell lines and vector control were cultured with no drug, imatinib (1 µM), dasatinib (10 nM), or nilotinib (50 nM) and the absolute number of Ki67-negative cells determined at 72 hours (a time point chosen to identify cells which were truly quiescent and not transiently arrested before dying). While treatment of vector control with each TKI significantly increased the number of Ki67-negative cells, over-expression of FOXO3a TM alone was sufficient to induce maximal fourfold increase in quiescent K562 cells, with no additional increase from the three TKIs (Fig. 6F, *, *p* < .05; **, *p* < .01). Although FOXO3a WT significantly increased the number of quiescent cells by only approximately threefold over vector control, there was no significant further increase with TKI. These data suggest that the vast majority of the antiproliferative effects of these agents are likely to sit on the same pathway as FOXO proteins. We had previously established that gene targets of FOXO3a including CCND1/Cyclin D1 and p57/CDKN1C, among others, were modulated upon treatment of CD34^+^ cells with TKIs. To determine whether this was also the case in our over-expression model, we measured mRNA expression levels of CCND1/Cyclin D1 and p57/CDKN1C. The FOXO3a TM cells showed a significant decrease in CCND1/Cyclin D1 levels (Fig. 5G, Left), although no increase in p57/CDKN1C (Fig. 6G, right; ***, *p* < .001; *n* = 3).

### Knockdown of FOXO3a Drives Ph^+^ Cells into Cycle and Co-Operates with Dasatinib to Induce Their Apoptosis

**Figure 7:**
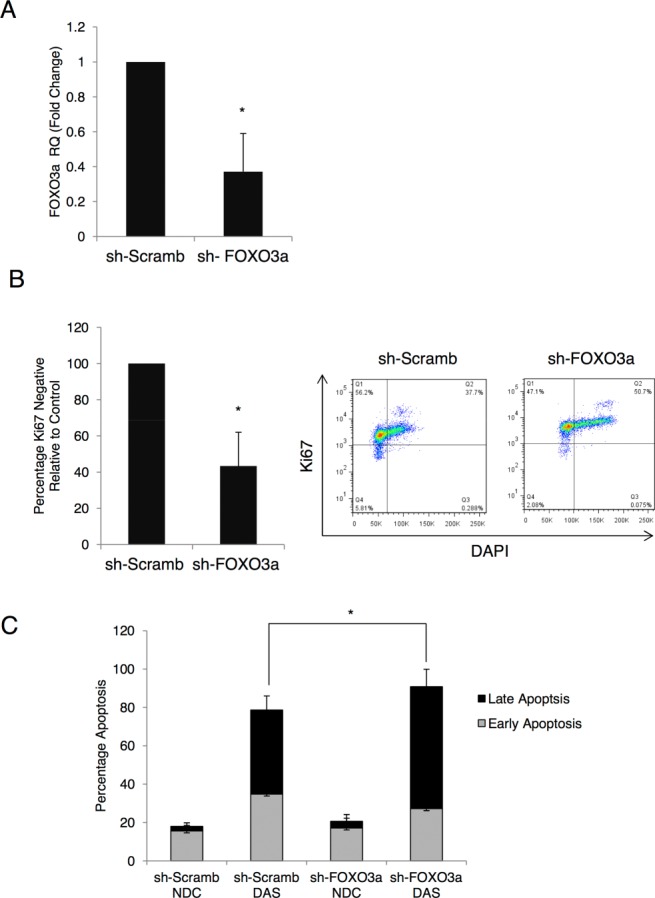
FOXO3a knockdown decreases quiescence and increases sensitivity to tyrosine kinase inhibitors (TKIs). **(A):** K562 cells were transfected with a FOXO3a shRNA plasmid containing a green fluorescent protein (GFP) tag (sh-FOXO3a) or a scrambled control (sh-Scramb). Twenty-four hours after transfection, GFP-positive cells were selected and knockdown of FOXO3a was confirmed by quantitative PCR (*n* = 3, *, *p* ≤ .05). **(B):** To determine the effect on sensitivity to TKIs, GFP-sorted cells were treated with dasatinib (DAS, 10 nM) for 72 hours and the level of apoptosis measured by Annexin V/DAPI staining (*n* = 3, *, *p* ≤ .05). **(C):** Staining with Ki67 and DAPI was used to determine the number Ki67 negative, quiescent cells following knockdown (left, *n* = 3, *, *p* ≤ .05). Representative dot-plots are shown (right). Abbreviations: DAS, dasatinib; DAPI, 4′6-diamidino-2-phenylindole; NDC, no drug control.

Having demonstrated over-expression of FOXO3a is sufficient to induce quiescence in Ph^+^ cells we assessed FOXO3a as a potential therapeutic target. K562 cells were transiently transfected with a GFP containing vector expressing either shRNA against FOXO3a or scrambled control. After 24 hours, cells were sorted for GFP expression and treated with and without 10 nM dasatinib for 72 hours. Q-PCR was used to confirm knockdown in GFP selected cells (Fig. 7A,*, *p* < .05). Given that over-expression of active FOXO3a was able to induce quiescence, we first wished to address whether in the absence of TKIs, inhibition of FOXO3a was able to drive cells into cycle. Ki67 and DAPI staining were used to quantify the number of quiescent cells in the cultures. Ki67-negative cells were reduced by 57% following FOXO3a knockdown (Fig. 7B, left; representative dot plots, right), confirming that inhibition of FOXO signaling was sufficient to drive quiescent cells into cycle (*, *p* < .05). To determine how loss of FOXO3a would affect sensitivity of the cells to TKIs, GFP-selected cells were incubated with and without 10 nM dasatinib for 72 hours and apoptosis determined using Annexin V/7-AAD staining. While knockdown alone did not increase apoptosis, in the presence of TKIs, the percentage of late apoptotic cells increased significantly and there was a trend toward an increase in total apoptosis (*, *p* < .05; *n* = 3) (Fig. 7C). The enhanced apoptosis with dasatinib might be explained by enhanced cytotoxicity of cells in active cycle.

## Discussion

CML is one of the few malignancies in which it has been possible to identify a plausible targeted therapy. Over the last 15 years, the development of TKIs has improved management for many CML patients 2. However, two key issues have been identified with their use. First, CML stem/progenitor cells are much less sensitive to apoptosis induction by TKIs as compared to more mature cells, likely explained by their lack of dependence on BCR-ABL kinase activity for survival 9,10. Second, TKIs exert potent antiproliferative effects against CML stem/progenitor cells that lead to a state of “induced quiescence.” This likely protects the cells from genomic instability, known to be induced by BCR-ABL, and may explain why few patients on TKIs progress to advanced phase disease. At the same time, the potential drawback is that quiescence makes the cells far more difficult to target from a therapeutic point of view.

BCR-ABL signaling activates the PI3K/AKT pathway (among others) 38, leading to inhibition of FOXO transcriptional activity 21. FOXO members play a role at the G0-G1, G1-S, and G2-M checkpoints via transcriptional modulation of proteins that regulate these transitions, thereby inducing cell cycle arrest. Our study demonstrates that the antiproliferative activity of TKIs against primary CML CD34^+^ cells is likely mediated by the reactivation of FOXO1, 3a, and 4. Previous reports in cell lines have shown that imatinib can rescue BCR-ABL-dependent inhibition of FOXO TFs 21,22; however, this is the first report that elucidates the role of FOXO TFs in CML versus normal human hemopoietic cells, including CD34^+^ cells. It also mechanistically demonstrates that FOXO TFs underlie the antiproliferative activity of TKIs, both in vitro and in an in vivo CML mouse model.

We have shown that in primary CML cells, BCR-ABL regulates FOXO1, 3a, and 4 at the post-transcriptional level, similar to previous observations 23. In CML CD34^+^ cells BCR-ABL expression leads to an increase of FOXO3a in the cytoplasm, where it is transcriptionally inactive, whereas in normal CD34^+^ cells, FOXO3a is predominantly detected in the nucleus. Together with FOXO3a, FOXO1 and 4 are also highly phosphorylated in CD34^+^ CML cells. Inhibition of BCR-ABL by TKIs reduces phosphorylation of FOXO TFs, drives their relocalization to the nucleus, and restores their transcriptional activity. Full restoration of FOXO activity in primary CML CD34^+^ cells correlated with a dramatic decrease in CCND1/Cyclin D1 mRNA level, and modulation of key FOXO target genes, ATM, p57/CDKN1C, and BCL6, which are all required for maintenance of HSC/LSCs 18,35,36. Similar results were observed in an in vivo CML model. Here, treatment of the leukemic mice with dasatinib for 6 days caused activation of FOXO1 and FOXO3a, as suggested by their phosphorylation status and by a marked decrease in CCND1/Cyclin D1 mRNA level. In addition, analysis of the transcriptional profile of CD34^+^ cells derived from CML patients treated with imatinib showed a regulation of the canonical FOXO target genes, including p57/CDKN1C and BCL6, suggesting that activation of FOXOs upon TKI treatment also occurs in humans in vivo. Although these results are indicative, a more thorough investigation would be required to definitively define the role of FOXOs in TKI-mediated G1 arrest in patients.

It has been suggested that upregulation of BCL6 by TKIs is responsible for the maintenance of CML stem cells through FOXO3a signaling and by repressing Arf and p53 18. These data, together with our findings, provide evidence that BCL6 and FOXO3a indeed function to protect CML stem cells from TKI treatment. Interestingly, Naka et al. showed that FOXO3a is essential for the maintenance of CML stem cells and suggested that TGF-β-mediated suppression of AKT activity resulted in increased levels of FOXO activity, positioning the role of TGF-β directly up-stream of the FOXO/BCL6 axis 16,19. Our study provides further insight into this critical signaling cascade leading from TGF-β through AKT to FOXO3a/BCL6/p53/ that maintains the survival of CML stem cells upon treatment with TKIs.

Although FOXO TFs themselves may not represent straight forward drug targets, the accumulating evidence suggests that the signaling network involving BCR ABL/PI3K/AKT/mTOR/FOXO/BCL6 is critical for the quiescence and survival of CML stem/progenitor cells and may be targeted for therapy. Naka et al. has already combined LY364947-mediated TGF-β inhibition with TKI treatment and found that CML was eradicated in the CML mouse model 16 and Hurtz et al. inactivated BCL6 using a retro-inverso BCL6 peptide inhibitor, resulting in delayed progression of CML when combined with TKIs 18.

In Ph^+^ cells, over-expression of WT or the activating mutant form of FOXO3a, FOXO3a TM, resulted in decreased proliferation and induction of apoptosis, effects not enhanced by dasatinib treatment. Furthermore, stable over-expression of FOXO3a TM increased the percentage of quiescent Ki67-negative cells to a level that was not further enhanced by addition of imatinib, dasatinib, or nilotinib. These data suggest that FOXO reactivation accounts for TKI-induced quiescence in CML cells. These results with FOXO3a over-expression were then complemented by results using inhibitors of the BCR-ABL/PI3K/AKT/mTOR pathway. Inhibition of mTOR signaling by rapamycin can either activate or inactivate the function of FOXO TFs in a cell context-dependent fashion 30, whereas LY294002 is an inhibitor of PI3K. In our hands, these agents and dasatinib produced very similar levels of inhibition of phosphorylation of FOXO TFs. Here, we used shRNA against FOXO3a in combination with TKIs to demonstrate that inhibition of FOXO signaling drives CML cells into cell cycle allowing them to undergo apoptosis in response to TKIs.

Finally, we have observed that in the most primitive CML stem cells (CD34^+^38^−^90^+^ and Lin^−^CD34^+^38^−^), FOXO TFs are less inhibited despite the presence of BCR-ABL (Supporting Information Fig. S5). These data for human CML stem cells mirror the work of Naka et al. in the CML mouse model 16. Although we have previously shown that CD34^+^38^−^ CML cells express high levels of BCR-ABL 12, our current data either suggest that BCR-ABL is not fully active in this population of primitive cells or that FOXO activity provides the dominant signal, thus providing an explanation for their intrinsic quiescence. These data suggest that treatment with TKIs of already quiescent CML stem cells may not have the same effect observed in progenitor cells. However, targeting the signaling network required for TKI-induced arrest in progenitor cells may also have an effect on the primitive TKI-insensitive cells, assuming a shared mechanism of regulation.

## Conclusion

Taken together these data confirm that by inhibiting phosphorylation of FOXO TFs, TKIs lead to their transcriptional reactivation, providing a valid explanation for the TKI-mediated G1 arrest observed in CML cells. In agreement with these data, the finding that the FOXO family plays a fundamental role in normal HSC maintenance makes them excellent candidates to explain our observations in leukemia 39. Our data on human CML cells greatly enhance our understanding of the regulation of quiescence in CML stem and progenitor cells and provide a platform from which to aim to manipulate stem cell quiescence for therapy.

## Supporting Information

See http://www.StemCells.com for supporting information available online.
