# Supplementary figures and images for: The Antiproliferative Activity of Kinase Inhibitors in Chronic Myeloid Leukemia Cells Is Mediated by FOXO Transcription Factors
Source: Stem Cells. 2014 Aug 18;32(9):2324–37. doi: 10.1002/stem.1748 (PMC4282530; doi:10.1002/stem.1748)

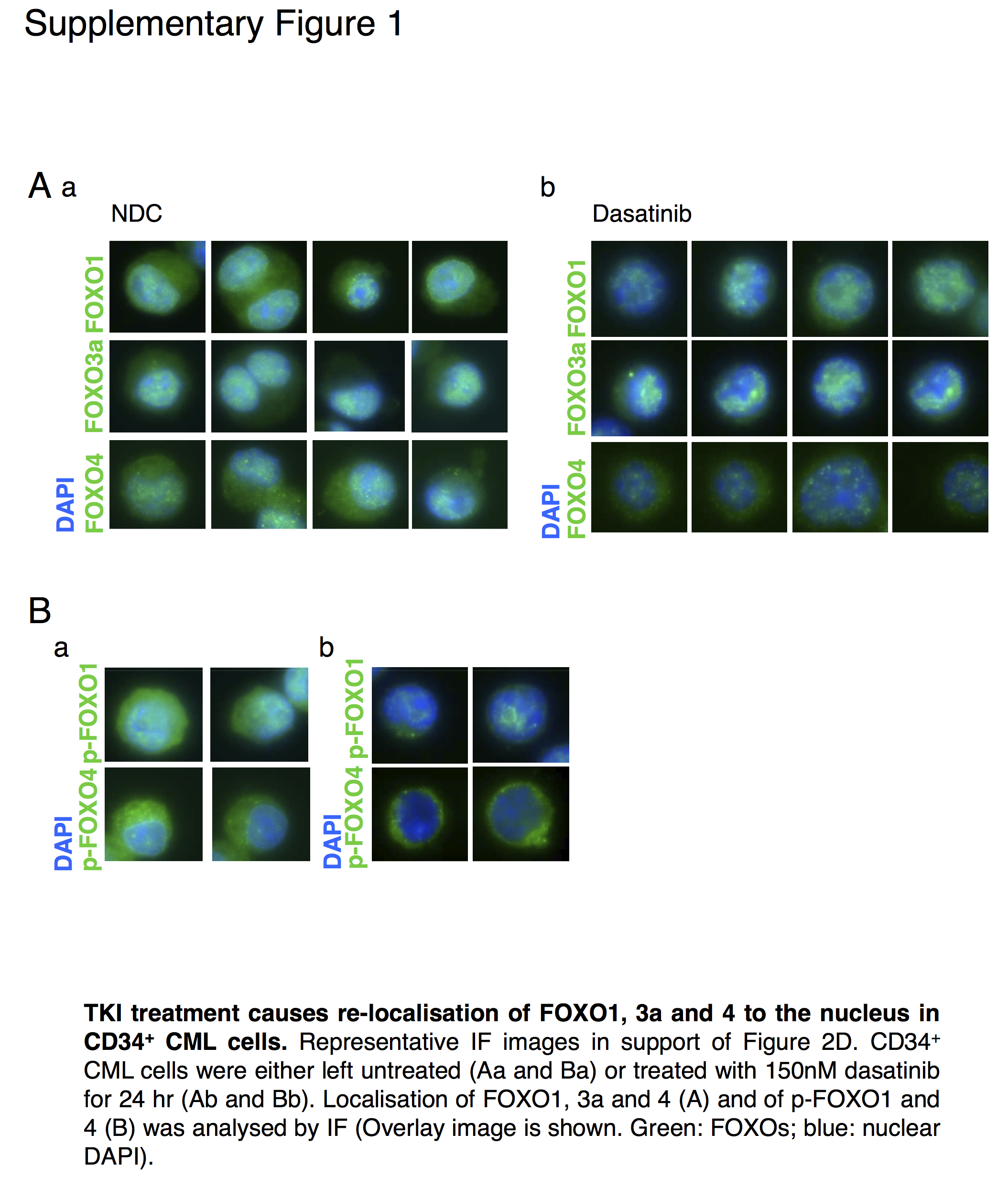

Supplement: Supplementary file 1 [file stem0032-2324-SD1.tiff]

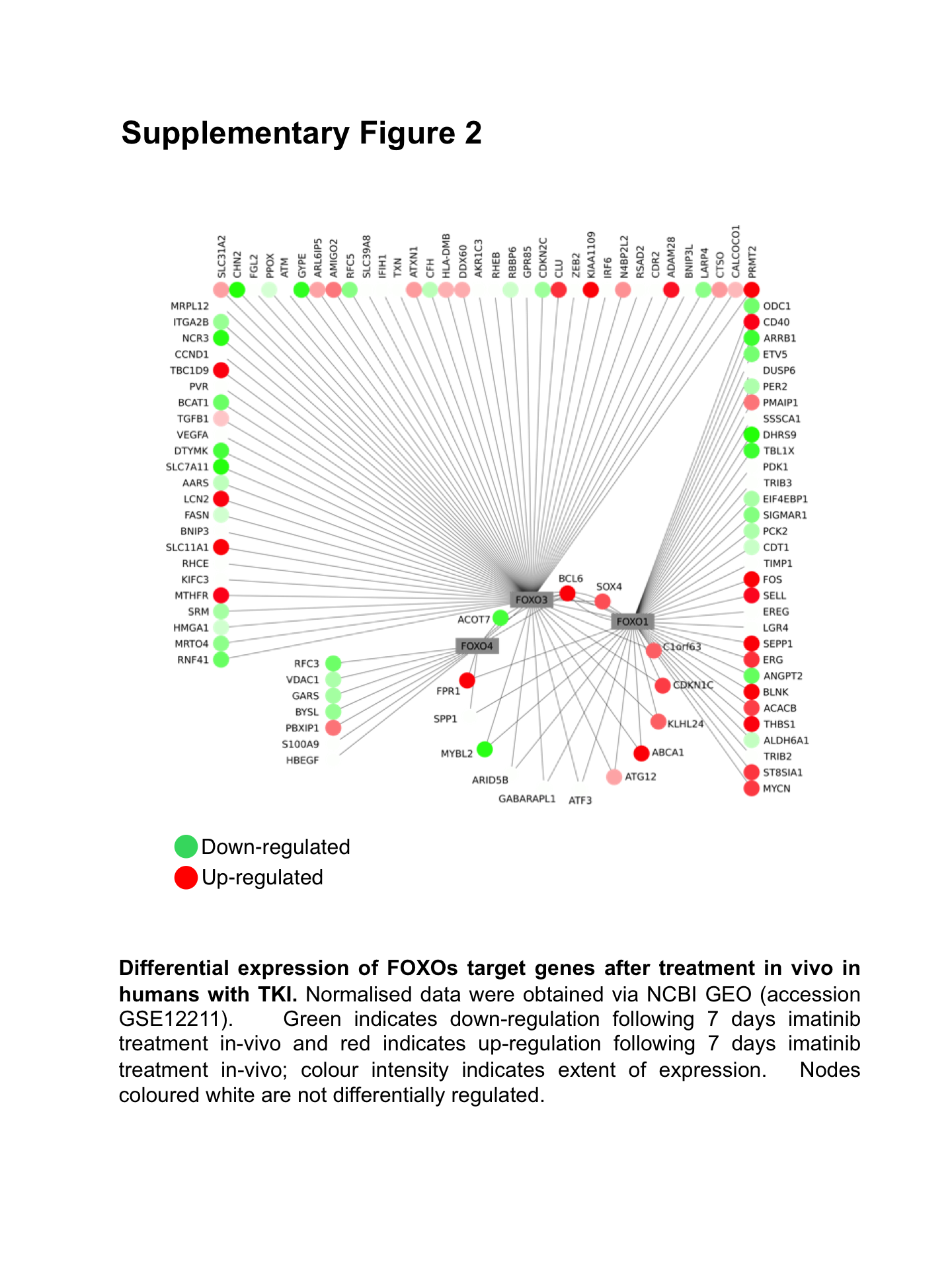

Supplement: Supplementary file 2 [file stem0032-2324-SD2.tiff]

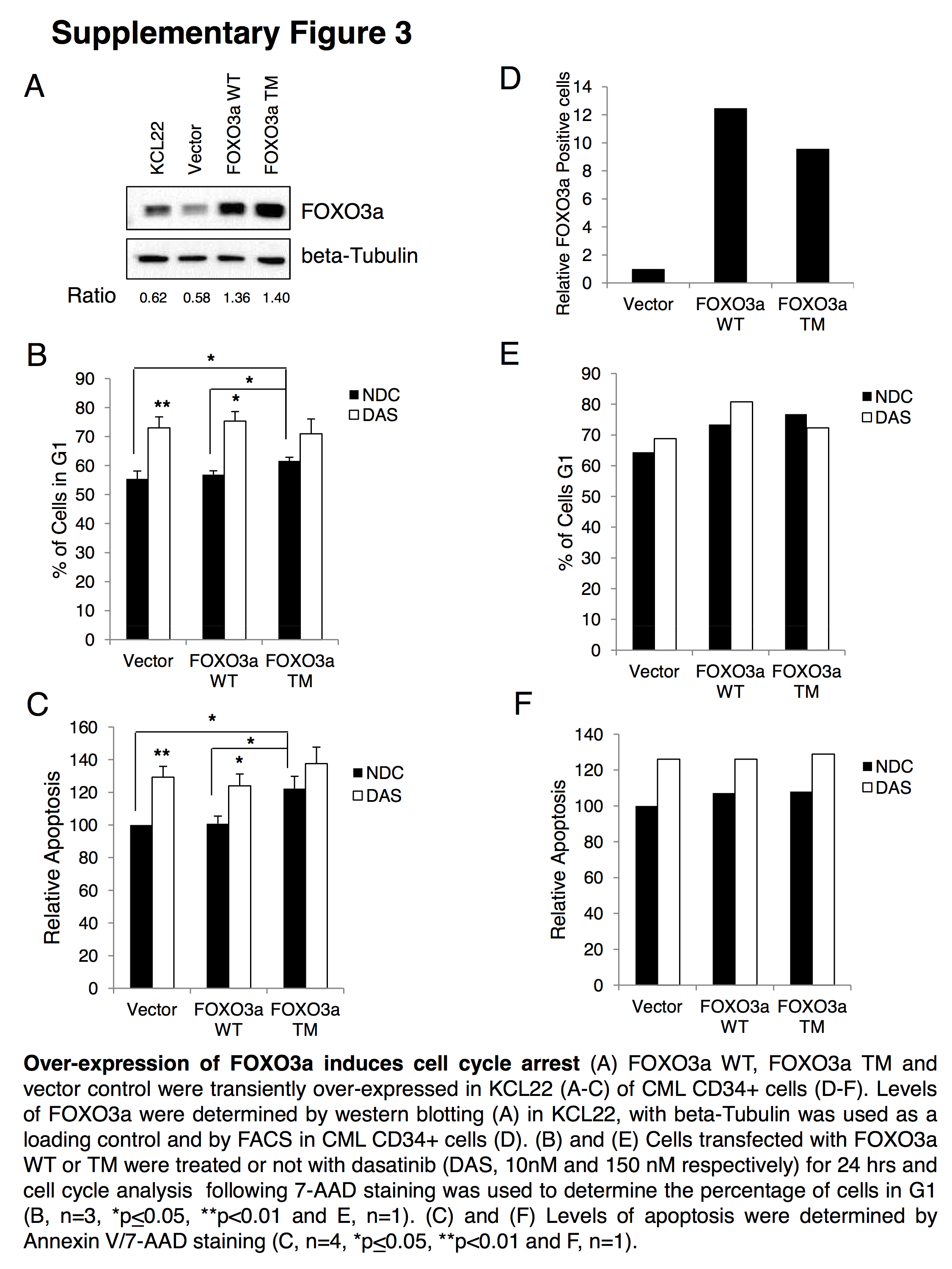

Supplement: Supplementary file 3 [file stem0032-2324-SD3.tiff]

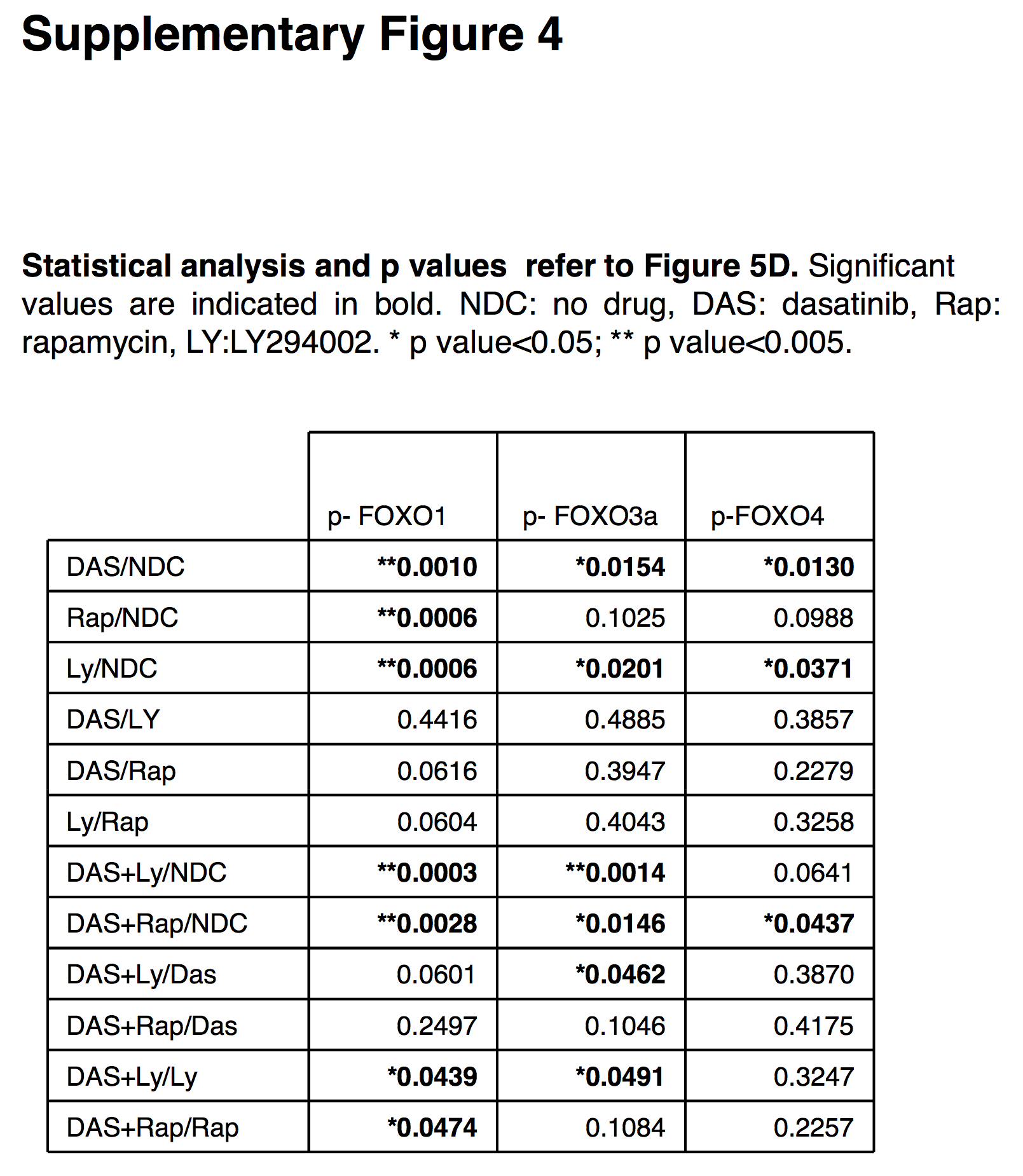

Supplement: Supplementary file 4 [file stem0032-2324-SD4.tiff]

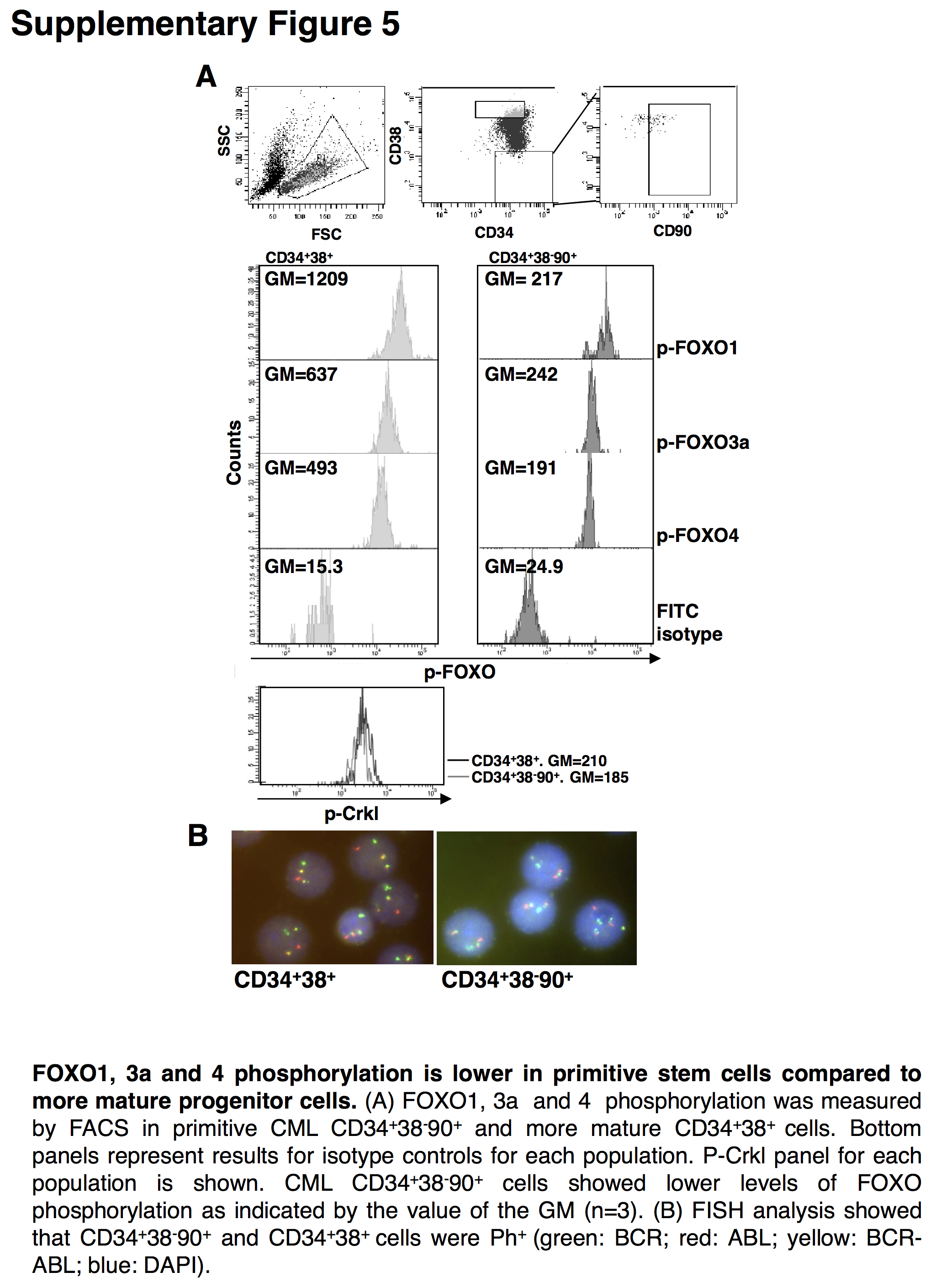

Supplement: Supplementary file 5 [file stem0032-2324-SD5.tiff]
